# Organizational management of moderate and severe paediatric traumatic brain injury: results from a European survey

**DOI:** 10.1016/j.bas.2025.105921

**Published:** 2026-01-19

**Authors:** Sarah Hornshøj Pedersen, Radek Frič, Shruti Agrawal, Chiara Robba, Aurelia Peraud, Miroslav Gjurasin, Ondra Petr, Marianne Juhler, Bart Depreitere

**Affiliations:** a Department of Neurosurgery, Copenhagen University Hospital, Copenhagen, Denmark; b Department of Neurosurgery, Oslo University Hospital-Rikshospitalet, Oslo, Norway; c Department of Paediatrics, University of Cambridge, Cambridge, UK; d IRCCS Policlinico San Martino, Genova, Italy; e Department of Neurosurgery, Section Pediatric Neurosurgery, University Hospital Ulm, Ulm, Germany; f Department of Neurosurgery, Children's Hospital Zagreb, Zagreb, Croatia; g Department of Neurosurgery, Medical University Innsbruck, Tyrol, Austria; h Department of Neurosurgery, Aarhus University Hospital, Aarhus, Denmark; i Neurosurgery, University Hospitals Leuven, Leuven, Belgium

**Keywords:** Paediatric traumatic brain injury, Moderate traumatic brain injury, Severe traumatic brain injury, Organizational management, Pediatric neurosurgery, Pediatric intensive medicine

## Abstract

**Introduction:**

Management of moderate/severe paediatric traumatic brain injury (mspTBI) varies across Europe. The decline in case numbers perceived in many regions raises concerns about maintaining high-quality, sustainable care.

**Research question:**

This study aimed to examine the organization of mspTBI management in Europe, focusing on expertise availability, guideline adherence, neuromonitoring use, and clinician's confidence in care delivery through a survey.

**Material and methods:**

A 34-question survey was distributed to European neurosurgical and intensive care communities. Only hospitals treating children with mspTBI were included. Centres were stratified by their catchment population size and their access to dedicated expertise.

**Results:**

Seventy-six institutions from 23 countries responded. Most centres reported a mspTBI case load of less than 20 children per year. Access to paediatric anesthesiology was significantly associated with centre size (p = 0.001), while access to paediatric neurosurgery, intensive or neurointensive care was not. Most centres (96 %) reported adherence to (inter)national guidelines. Intracranial pressure (78.7 %) and transcranial Doppler (70.7 %) were the most frequently available/used neuromonitoring modalities. Confidence in managing mspTBI was significantly higher in centres with paediatric neurosurgeons and, for older children, paediatric neuro-intensivists.

**Discussion:**

This first European survey examining organizational management of mspTBI reveals a low overall caseload and uneven access to paediatric expertise. Confidence in managing mspTBI correlates with availability of paediatric subspecialists. Guidelines are widely applied, independent of expert availability, but are alone insufficient to ensure treatment confidence. This finding underscores the need for improved guidelines and better access to paediatric neurotrauma expertise.

## Highlights

• Organization of management of moderate to severe paediatric traumatic brain injury in Europe was examined through a survey among European neurosurgical and intensive care communities.
• Most centres reported a case load of moderate to severe paediatric traumatic brain injury of less than 20 children per year.
• Confidence in managing moderate to severe traumatic brain injury in children correlates with availability of paediatric subspecialists.

## 1 Introduction

Paediatric traumatic brain injury is a major contributor to morbidity and mortality in children worldwide. More seriously injured children, those with moderate to severe traumatic brain injury (mspTBI), have a profound impact on healthcare systems, families, and society (Dewan et al., 2016). While the incidence of mspTBI in children appears to be declining in most high-income countries, including Europe (Guan et al., 2023; Dahl et al., 2021; Maas et al., 2022; Feigin et al., 2021), it remains a high-impact condition requiring prompt, resource-intensive, and often prolonged multidisciplinary care. Optimal management of mspTBI involves coordinated neurocritical care, neurosurgical expertise and paediatric-specific rehabilitation.

There are several different models of practice in the management of mspTBI, with significant variations among countries and in some cases also within the same country (Wickbom et al., 2022; Chung et al., 2024). It is not clear how these variations affect the quality of care for these patients. With the decline in incidence of severely injured children, many centres may find themselves managing fewer cases annually. This trend, although positive, raises critical questions about how to best maintain clinical expertise, allocate resources efficiently, and design models of care that are both effective and sustainable. In particular, understanding current hospital capabilities, paediatric subspecialist availability as well as the requirements for best patient outcomes are important.

Despite the clinical importance of these issues, published data on infrastructure and access to care for mspTBI remain limited in the European context (Bruns et al., 2021; Trefan et al., 2016; Jochems et al., 2021). Further, there is limited consensus on how services should be organized and how clinical expertise can be maintained in the context of decreasing caseloads. To address this gap, we conducted a survey describing the organization and practices of mspTBI management across different European countries.

## 2 Methods

### 2.1 Population

The target population consisted of 1) the general neurosurgical community in EANS (European Association of Neurosurgical Societies) member countries, 2) the paediatric intensive care community in ESPNIC (European Society of Pediatric and Neonatal Intensive Care) member countries, and 3) the general intensive care community in ESCIM (European Society of Intensive Care Medicine) member countries. All individual members of the three societies were invited to participate in the survey.

To collect a representative dataset without multiple entries from the same institution, we asked the respondents (neurosurgeons and intensive care specialists) to submit only one survey on behalf of their institution. Duplicates and incomplete surveys were removed from the dataset after clarifying with the respondents. Non-European hospitals and hospitals only treating adults were excluded. There were no additional exclusion criteria. The information provided was treated confidentially and, after closing the dataset, analyzed in an anonymized manner.

### 2.2 Design

The survey was designed and elaborated in a working group consisting of the authors representing all three scientific societies. Before circulating the questionnaire to members of the EANS, ESPNIC and ESCIM, it was tested, approved and endorsed by all three societies. The survey consisted of 34 questions divided into seven main sections: A) Hospital setting and resources; B) Management of mspTBI in the hospital, distinguishing between children (2–18 years of age) and infants (0–2 years of age); C) Specialist in-charge of mspTBI; D) Availability of paediatric subspecialty expertise; E) Rehabilitation and follow-up; F) Registry; and G) Opinion (the full questionnaire is provided in Supplementary material).

The 34 survey questions generated a large number of answers, from which we selected four focus areas. Within each focus area, one indicator question was selected based on content matching of the focus area, the clarity of the data (i.e., the answer options) and completeness of answers.

### 2.3 Statistical analysis

We used a stratified approach subgrouping participating centres firstly according to catchment population size (<0.5 million, 0.5–1 million and >1 million), and secondly according to access to subspecialty expertise in paediatric neurosurgery and paediatric intensive care. This strategy was based on the underlying hypothesis that availability of dedicated expertise is related to the size of the catchment population. We subsequently investigated whether access to such expertise in mspTBI care was associated with.

• adherence to paediatric TBI guidelines
• use of invasive intracranial (ICP) monitoring
• use of multimodal monitoring
• clinician confidence in treatment of children and infants with moderate/severe TBI

Expert availability was defined as the presence of 24/7 coverage, either through a formally established on-call service or availability without formal on-call arrangements. Access was stratified across four paediatric subspecialist domains: neurosurgery, intensive care, neuro-intensive care, and anaesthesia. For analysis, availability within each domain was dichotomized (yes/no). The questionnaire further distinguished between the Scandinavian guidelines for initial management of minor and moderate head trauma in children, 2016 (Astrand et al., 2016); Italian guidelines on the assessment and management of pediatric head injury in the emergency department, 2018 (Da Dalt et al., 2018); Guidelines for the management of pediatric severe traumatic brain injury, 3rd edition, Update of the Brain Trauma Foundation, 2019 (Kochanek et al., 2019); The management of pediatric severe traumatic brain injury: Italian Guidelines, 2021 (Bussolin et al., 2021), and other national or local child-specific guidelines. However, as the recommendations across these are essentially similar, we did not differentiate them in the analysis.

Data are presented as medians and percentages. Centres are compared using Fisher's exact test due the relatively small number of centres in each group. Statistical analyses were conducted in R Studio v 2024.04.2.

## 3 Results

**Fig. 1:**
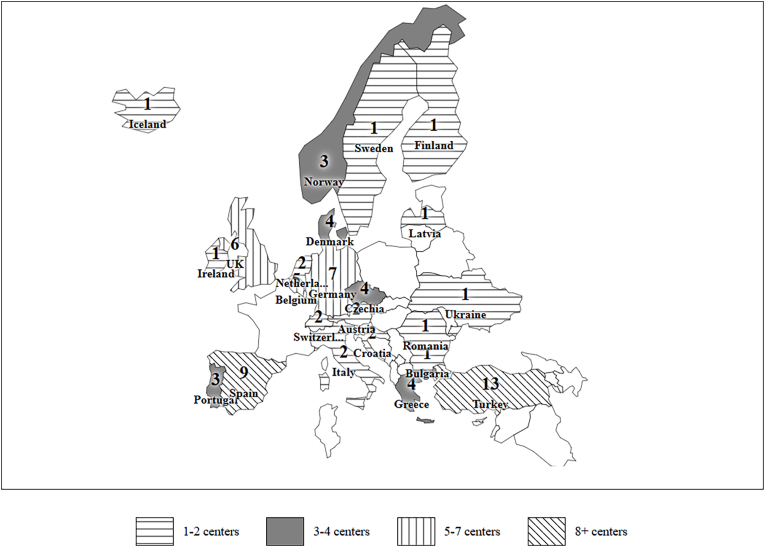
Geographical distribution of centres participating in the survey.

An overview of participating countries with total estimated paediatric population size as well as number of included centres per country and their characteristics are shown in Fig. 1and Table 1, Table 2. After removal of duplicate institutions and non-European respondents, there were 76 separate entries (institutions) from 23 countries across Europe. The majority were academic and Level 1 trauma centres. More than three quarters of the respondents (n = 59; 78 %) treated both adult and paediatric TBI, while only 17 respondents (22 %) were children-only institutions. One respondent reported treating only adult patients and was thus excluded.

**Table 1:** Demographics of the participating countries.

Country	Estimate of total population size∗	Percent of total population under age 18 year [%]	Percent of total population under age 2 year [%]	Number of centres responded to the survey
Austria	8,980,000	19.4	2.1	2
Belgium	11,500,000	21.3	2.4	5
Bulgaria	6,800,000	17.6	2.0	1
Croatia	3,900,000	18.5	2.1	2
Czechia	10,700,000	19.0	2.2	4
Denmark	5,890,000	20.3	2.2	4
Finland	5,540,000	19.2	2.0	1
Germany	83,100,000	18.1	2.0	7
Greece	10,400,000	18.4	2.1	4
Iceland	375,000	20.1	2.2	1
Ireland	5,100,000	22.0	2.3	1
Italy	59,000,000	17.7	1.9	2
Latvia	1,860,000	18.0	2.0	1
Netherlands	17,400,000	22.0	2.5	2
Norway	5,420,000	21.5	2.4	3
Portugal	10,300,000	19.7	2.1	3
Romania	19,000,000	20.0	2.3	1
Spain	47,400,000	19.0	2.0	9
Sweden	10,400,000	20.3	2.1	1
Switzerland	8,900,000	18.5	2.0	2
Turkey	86,900,000	27.2	3.0	13
Ukraine	37,000,000	22.1	2.4	1
United Kingdom	68,000,000	20.6	2.3	6

Number of participating centres per country, in addition to the total population size and percentage children and infants per country (DelSignore and Tasker, 2017).

**Table 2:** Characteristics of participating hospitals.

Country	Hospital type (mixed children/adults or children only)	Trauma Centre Level	Population size
Austria	mixed	level 1	>1,000,000
	mixed	level 1	500,000–1,000,000
Belgium	mixed	level 1	<500,000
	mixed	level 1	>1,000,000
	mixed	level 2	<500,000
	mixed	level 1	500,000–1,000,000
	mixed	not applicable	<500,000
Bulgaria	mixed	level 1	500,000–1,000,000
Croatia	children	level 1	>1,000,000
	mixed	level 1	>1,000,000
Czechia	mixed	level 1	>1,000,000
	mixed	level 1	500,000–1,000,000
	mixed	level 1	500,000–1,000,000
	mixed	level 1	>1,000,000
Denmark	mixed	level 1	>1,000,000
	mixed	level 1	500,000–1,000,000
	mixed	level 1	>1,000,000
	mixed	level 1	>1,000,000
Finland	children	level 1	>1,000,000
Germany	mixed	level 1	500,000–1,000,000
	children	level 1	>1,000,000
	mixed	level 1	>1,000,000
	mixed	level 1	500,000–1,000,000
	children	level 1	<500,000
	mixed	level 1	>1,000,000
	mixed	level 1	500,000–1,000,000
Greece	mixed	level 3	>1,000,000
	mixed	level 1	500,000–1,000,000
	children	level 1	>1,000,000
	mixed	not applicable	<500,000
Iceland	mixed	level 1	<500,000
Ireland	children	level 1	>1,000,000
Italy	children	level 1	500,000–1,000,000
	children	level 1	>1,000,000
Latvia	children	not applicable	<500,000
Netherlands	mixed	level 1	>1,000,000
	mixed	level 1	<500,000
Norway	mixed	level 3	>1,000,000
	mixed	level 1	500,000–1,000,000
	mixed	level 2	500,000–1,000,000
Portugal	mixed	level 1	>1,000,000
	children	level 1	>1,000,000
	children	level 2	>1,000,000
Romania	mixed	level 1	<500,000
Spain	mixed	level 1	>1,000,000
	mixed	level 1	500,000–1,000,000
	mixed	level 1	<500,000
	children	level 1	>1,000,000
	mixed	level 1	<500,000
	mixed	level 1	>1,000,000
	mixed	level 1	<500,000
	mixed	level 1	>1,000,000
	mixed	level 1	>1,000,000
Sweden	mixed	level 1	>1,000,000
Switzerland	children	level 1	>1,000,000
	children	level 1	<500,000
Turkey	mixed	level 2	>1,000,000
	mixed	not applicable	>1,000,000
	mixed	level 1	500,000–1,000,000
	mixed	level 2	>1,000,000
	children	level 1	<500,000
	mixed	Level 2	<500,000
	mixed	not applicable	<500,000
	mixed	not applicable	>1,000,000
	mixed	level 3	>1,000,000
	mixed	level 2	<500,000
	mixed	not applicable	<500,000
	mixed	level 1	>1,000,000
	mixed	not applicable	<500,000
Ukraine	mixed	level 1	500,000–1,000,000
United Kingdom	children	level 1	500,000–1,000,000
	mixed	level 1	>1,000,000
	mixed	level 1	>1,000,000
	mixed	level 1	>1,000,000
	children	level 1	>1,000,000
	mixed	level 1	>1,000,000

Hospital characteristics in terms of paediatric vs. mixed service, trauma centre level and catchment population size.

Trauma centre Level 1: most comprehensive trauma care; Level 2: almost full coverage 24 h; Level 3: no 24 h and no full coverage.

### 3.1 Case load and expertise availability

**Fig. 2:**
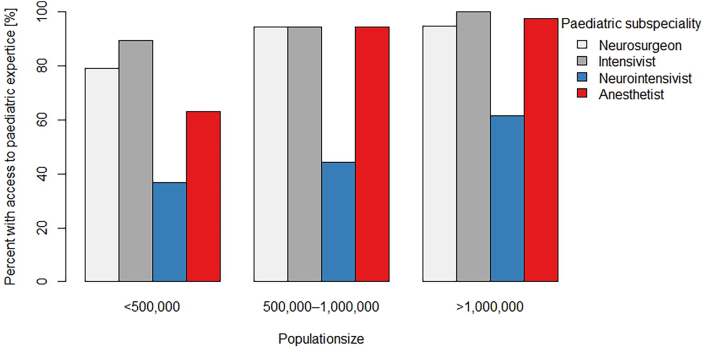
Availability of paediatric subspecialist care (for neurosurgery, intensive care, neurointensive care and anesthesiology) in participating hospitals in relation to catchment area population.

More than half of the institutions (n = 46; 61 %) reported an annual case load of less than 20 mspTBI in the 2–18 years group, while 87 % (n = 66) reported similar annual case load in the 0–2 years group. The reported median full time equivalent (FTE) specialists per centre were 10 (range 2–50) for neurosurgery and 15 (range 1–140) for intensive care. For both specialties, the median FTE of specialists active in the paediatric subspecialty for more than 50 % of their practice was 2 and 6, respectively. Availability of dedicated paediatric subspecialty expertise relevant for mspTBI is shown in Fig. 2. Availability of pediatric anesthesiology was significantly higher in larger centres (p = 0.001). However, availability of paediatric neurosurgery (p = 0.132), paediatric intensivist (p = 0.111) or paediatric neurointensivist (p = 0.234) was not associated with the centre size. Thirty-six institutions (47 %) reported a 24/7 formal on-call service for paediatric neurosurgery, 65 (86 %) for paediatric intensive care, 20 (26 %) for paediatric neurointensive care and 53 (70 %) for paediatric anaesthesia. Most institutions (72; 95 %) reported access to rehabilitation services following mspTBI, with 51 (67 %) reporting dedicated paediatric rehabilitation.

### 3.2 Adherence to guidelines

Most respondents (n = 73; 96 %) reported the use of national or international guidelines for managing mspTBI. Of these, 68.5 % applied a single guideline (n = 50), while 24.7 % used two (n = 18). Among respondents using one guideline, the most frequently applied was the *Brain Trauma Foundation Guidelines for the Management of Pediatric Severe Traumatic Brain Injury* (3rd edition, 2019), used by 41 of 50 respondents (82 %). For those using two guidelines (n = 18), the Brain Trauma Foundation guidelines were included in 17 cases, and were in 11 of these supplemented by a national or local guideline.

Overall, 63 respondents used the Brain Trauma Foundation guidelines, 22 reported using local or national guidelines, 13 used the *Scandinavian guidelines for initial management of minor and moderate head trauma in children* (2016), 4 used *The management of pediatric severe TBI: Italian guidelines* (2021), and 1 used the *Italian guidelines on the assessment and management of pediatric head injury in the emergency department* (2018). Guideline use was independent of 24/7 availability of expertise in the four paediatric subspecialist domains [neurosurgery (p = 1.0), intensive care (p = 1.0), neuro-intensive care (p = 0.605) and anesthesiology (p = 0.319)].

### 3.3 ICP monitoring and multimodal monitoring

**Fig. 3:**
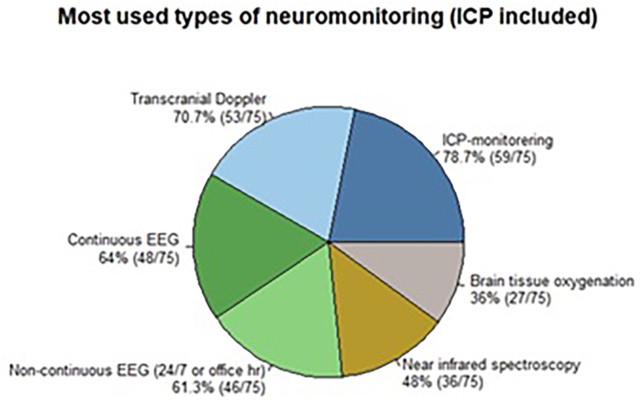
The six most frequently reported types of neuromonitoring.

The distribution of the six most commonly available/used neuromonitoring options is shown in Fig. 3. Invasive ICP and transcranial Doppler were the most frequently available/used monitoring methods utilized in 79 % and 71 % of the centres, respectively. Though multimodal monitoring was available/used in 74 centres (97 %), independent of access to paediatric expertise, centres with access to dedicated paediatric subspecialist expertise within the four domains seemed more likely to use ICP monitoring in managing the entire age-spectrum of mspTBI (Table 3).

**Table 3:** Use of neuromonitoring in children and infants with mspTBI.

**ICP monitoring in children age > 2 years old**		
*Access to neurosurgery*	*Use of ICP monitoring [%] (number of centres)*	*No use of ICP monitoring [%] (number of centres)*
Yes	81.2 % (56)	18.8 % (13)
No	57.1 % (4)	42.9 % (3)
*Access to intensive care*	*Use of ICP monitoring [%] (number of centres)*	*No use of ICP monitoring [%] (number of centres)*
Yes	79.5 % (58)	20.5 % (15)
No	66.7 % (2)	33.3 % (1)
*Access to neuro-intensive care*	*Use of ICP monitoring [%] (number of centres)*	*No use of ICP monitoring [%] (number of centres)*
Yes	87.2 % (34)	12.8 % (5)
No	69.4 % (25)	30.6 % (11)
*Access to anesthesiology*	*Use of ICP monitoring [%] (number of centres)*	*No use of ICP monitoring [%] (number of centres)*
Yes	80.6 % (54)	19.4 % (13)
No	66.7 % (6)	33.3 % (3)
**ICP monitoring in infants age < 2 years old**		
*Access to neurosurgery*	*Use of ICP monitoring [%] (number of centres)*	*No use of ICP monitoring [%] (number of centres)*
Yes	62.3 % (43)	37.7 % (26)
No	28.6 % (2)	71.4 % (5)
*Access to intensive care*	*Use of ICP monitoring [%] (number of centres)*	*No use of ICP monitoring [%] (number of centres)*
Yes	58.9 % (43)	41.1 % (30)
No	66.7 % (2)	33.3 % (1)
*Access to neuro-intensive care*	*Use of ICP monitoring [%] (number of centres)*	*No use of ICP monitoring [%] (number of centres)*
Yes	64.1 % (25)	35.9 % (14)
No	52.8 % (19)	47.2 % (17)
*Access to anesthesiology*	*Use of ICP monitoring [%] (number of centres)*	*No use of ICP monitoring [%] (number of centres)*
Yes	59.7 % (40)	40.3 % (27)
No	55.6 % (5)	44.4 % (4)
**Multimodal monitoring (not including ICP) in all pediatric patients age > 0 years old**		
*Access to neurosurgery*	*Use of multimodal monitoring [%] (number of centres)*	*No use of multimodal monitoring [%] (number of centres)*
Yes	98.6 % (68)	1.4 % (1)
No	100.0 % (7)	0.0 % (0)
*Access to intensive care*	*Use of multimodal monitoring [%] (number of centres)*	*No use of multimodal monitoring [%] (number of centres)*
Yes	98.6 % (72)	1.4 % (1)
No	100.0 % (3)	0.0 % (0)
*Access to neuro-intensive care*	*Use of multimodal monitoring [%] (number of centres)*	*No use of multimodal monitoring [%] (number of centres)*
Yes	100.0 % (39)	0.0 % (0)
No	97.2 % (35)	2.8 % (1)
*Access to anesthesiology*	*Use of multimodal monitoring [%] (number of centres)*	*No use of multimodal monitoring [%] (number of centres)*
Yes	98.5 % (66)	1.5 % (1)
No	100.0 % (9)	0.0 % (0)

Correlation between availability of paediatric subspecialty expertise within one of the four domains (neurosurgery, intensive care, neuro-intensive care and anesthesiology) and use of monitoring. P values were non-significant, ranging from 0.090 to 1. ICP = intracranial pressure. Multimodal monitoring includes continuous electroencephalogram (EEG), non-continuous EEG (24/7 available), non-continuous EEG (in office hours), amplitude integrated EEG, near infrared spectroscopy (NIRS), brain tissue oxygenation (PbtO2), microdialysis, transcranial Doppler, pressure reactivity index (PRx) and ‘other’.

### 3.4 Confidence in treating children and infants with mspTBI

Generally, respondents from centres with access to paediatric subspecialist expertise were more comfortable treating children and infants with mspTBI (Table 4). Availability of a paediatric neurosurgeon was significantly associated with the reported confidence to treat all or most children (p = 0.019) and all or most infants (p = 0.003). For children >1 year of age, availability of a paediatric neuro-intensivist was likewise correlated significantly with the reported confidence to treat most/all children (95 % vs 75 %; p = 0.018). Though similar raw data, the correlation was not significant for treating infants (90 % vs 78 %; p = 0.116).

**Table 4:** Confidence in managing children and infants with moderate/severe TBI.

Comfortable treating mspTBI	For no cases	For a minority of cases	For about half of cases	For most cases	For all cases	Not applicable
*Access to paediatric neurosurgery (p=0.019)*						
Yes	0.0 % (0)	1.4 % (1)	4.3 % (3)	23.2 % (16)	65.2 % (45)	5.8 % (4)
No	0.0 % (0)	14.3 % (1)	14.3 % (1)	42.9 % (3)	14.3 % (1)	14.3 % (1)
*Access to paediatric intensive care (p=1)*						
Yes	0.0 % (0)	2.7 % (2)	5.5 % (4)	26.0 % (19)	60.3 % (44)	5.5 % (4)
No	0.0 % (0)	0.0 % (0)	0.0 % (0)	0.0 % (0)	66.7 % (2)	33.3 % (1)
*Access to paediatric neurointensive care∗ 1 missing data (p=0.018)*						
Yes	0.0 % (0)	0.0 % (0)	2.6 % (1)	17.9 % (7)	76.9 % (30)	2.6 % (1)
No	0.0 % (0)	5.6 % (2)	8.3 % (3)	33.3 % (12)	41.7 % (15)	11.1 % (4)
*Access to paediatric anesthesiology (p=0.093)*						
Yes	0.0 % (0)	1.5 % (1)	4.5 % (3)	23.9 % (16)	64.2 % (43)	6.0 % (4)
No	0.0 % (0)	11.1 % (1)	11.1 % (1)	33.3 % (3)	33.3 % (3)	11.1 % (1)

Comfortable treating infants with msTBI	For no cases	For a minority of cases	For about half of cases	For most cases	For all cases	Not applicable
*Access to paediatric neurosurgery (p=0.0032)*						
Yes	0.0 % (0)	0.0 % (0)	4.3 % (3)	31.9 % (22)	56.5 % (39)	7.2 % (5)
No	14.3 % (1)	14.3 % (1)	14.3 % (1)	28.6 % (2)	14.3 % (1)	14.3 % (1)
*Access to paediatric intensive care (p=1)*						
Yes	1.4 % (1)	1.4 % (1)	5.5 % (4)	31.5 % (23)	53.4 % (39)	6.8 % (5)
No	0.0 % (0)	0.0 % (0)	0.0 % (0)	33.3 % (1)	33.3 % (1)	33.3 % (1)
*Access to paediatric neurointensive care∗ 1 missing data (p=0.117)*						
Yes	2.6 % (1)	0.0 % (0)	2.6 % (1)	25.6 % (10)	64.1 % (25)	5.1 % (2)
No	0.0 % (0)	2.8 % (1)	8.3 % (3)	38.9 % (14)	38.9 % (14)	11.1 % (4)
*Access to paediatric anesthesiology (p=0.057)*						
Yes	1.5 % (1)	0.0 % (0)	4.5 % (3)	29.9 % (20)	56.7 % (38)	7.5 % (5)
No	0.0 % (0)	11.1 % (1)	11.1 % (1)	44.4 % (4)	22.2 % (2)	11.1 % (1)

Graded response in confidence related to availability of dedicated paediatric subspecialty expertise in four domains (neurosurgery, intensive care, neuro-intensive care and anesthesiology).

mspTBI = moderate and severe paediatric traumatic brain injury.

For improving confidence in treating mspTBI, respondents mentioned specific management guidelines (43 centres (57 %) for children, 40 centres (53 %) for infants), option of tele-consultation with experienced paediatric neurosurgeons/intensivists (12 centres (16 %) each for children and infants), and a regionally organized stepped referral system (13 centres (17 %) for children and 12 centres (16 %) for infants).

## 4 Discussion

To the best of our knowledge, this is the first survey mapping the models of management of mspTBI in Europe. Our purpose was to explore the availability of dedicated paediatric expertise in managing mspTBI as well as the adherence to guidelines and clinician confidence in treating mspTBI.

Although the survey does not represent all relevant institutions or all EANS, ESPNIC and ESCIM member countries, the information gathered from this survey points out several interesting facts.

### 4.1 Case load of mspTBI

Overall, the annual number of mspTBI cases in responding European centres is low, but a few centres reported a much higher incidence. We are unable to assess whether this reflects truly higher numbers in some regions due to different epidemiology of mspTBI, higher grade of centralization, or whether the distinction between mild and moderate TBI is different among centres. Most academic large hospitals represented in this survey treat less than 20 children per year. These results align with the reported decline in mspTBI incidence in high income countries (Guan et al., 2023; Dahl et al., 2021; Maas et al., 2022; Feigin et al., 2021). This brings challenges for keeping up clinical experience in managing mspTBI.

### 4.2 Availability of expertise related to centre size

Our underlying hypothesis that availability of dedicated paediatric expertise could be related to centres' size and the size of population in their respective catchment area was supported by raw numbers for all four domains (neurosurgery, intensive and neurointensive care and anesthesiology), though a significant association could only be documented for availability of paediatric anesthesiology.

### 4.3 Use of neuromonitoring

Neuromonitoring is nearly universally available/used, with ICP being the most frequently monitored parameter, followed by transcranial Doppler. However, one quarter of the respondents did not use ICP monitoring, which can reflect the inclusion of cases with mild TBI that would also be an explanation for the large number of annual cases reported by some centres. It may also suggest that ICP monitoring is not utilized in some cases, in spite of the existing guidelines recommending this monitoring modality. Electroencephalogram (EEG) and near infrared spectroscopy (NIRS) were available/used by roughly half of the respondents, which could be explained by most participating centres being academic. Overall, the monitoring tools being available/used match well with current insights in recent publications on mspTBI management (Figaji, 2023; DelSignore and Tasker, 2017). Although statistical significance was not reached, likely due to small subgroup sizes, the data strongly suggests a disparity in the application of critical neuromonitoring tools for the most vulnerable age group in centres without dedicated neurosurgical expertise.

### 4.4 Adherence to guidelines

Almost all participating centres reported the availability and use of guidelines for the management of mspTBI, however, this was not entirely congruent with the clinician's reported levels of confidence. This discrepancy suggests a gap between the availability and application of guidelines, and their perceived practical usefulness. It is also reflected in the suggested ways to improve confidence**:** more specific management guidelines, tele-consultation with experienced paediatric neurosurgeons/intensivists and stepped referral systems. In addition, awareness of guidelines and their contents does not necessarily equate to structured implementation, which requires dedicated tools and frameworks (Pilar et al., 2020). Consequently, adherence to guidelines in paediatric TBI management remains low, with a recent study from the Netherlands estimating adherence rates between 39 and 65 % (Broers et al., 2018). Expertise and guidance should also extend into the post-acute phase of mspTBI, as almost half of affected children have unmet needs months after their trauma (Dahl et al., 2023).

### 4.5 Confidence in treating children and infants with mspTBI related to paediatric expert availability

A statistically significant association was observed between access to *paediatric neurosurgery* and confidence in treating both children and infants with similar trends observed for *paediatric neuro-intensivist* in treating children and *paediatric anesthesiology* in treating infants with mspTBI. Although some relationships were inconsistent, the overall data suggests that centres with any form of dedicated paediatric expertise tend to report a higher confidence in managing mspTBI.

While the survey does not directly address whether access to paediatric expertise improves outcome, the reported confidence in managing mspTBI may potentially serve as a surrogate measure for quality of care, or this should at least be investigated. The relationship between availability of relevant paediatric expertise and reported confidence supports this hypothesis. Establishing a causal link however, would require comparison of clinical outcomes related to expertise availability, which was not part of this survey.

Overall, the results of this survey indicate that the quality of care in children and infants with TBI could be improved by increasing access to at least one dedicated paediatric specialty relevant for mspTBI. Additionally, they also underscore the need to improve the practical usefulness of trauma guidelines. The finding that tele-consulting with experienced paediatric neurosurgeons/intensivists and a regionally organized stepped referral system scored rather low as options to improve confidence in treating mspTBI, is likely explained by the large majority of participants being tertiary centres, judging they should be the ones consulted or referred to. In other contexts and pathologies, tele-consulting has proven its worth as a viable and cost-effective strategy.

### 4.6 Limitations

Although the survey was broadly distributed to all EANS, ESPNIC and ESCIM members, more than 90 % of respondents defined their institution as an “academic hospital”. The survey results thus primarily reflect the organization, expertise availability and management policies at specialized tertiary centres and may not be representative for all hospital types. However, as mspTBI in Europe is rarely treated outside tertiary centres, the high proportion of academic hospitals reasonably reflects current clinical practice, thus limiting the possible selection bias. If not, note that this would even further strengthen the relevance of our findings, as this could mean that use of monitoring, guideline adherence and paediatric subspecialty availability is overestimated. To further contextualize representativeness, we have included general demographic data in Table 1 on total and paediatric population sizes in the participating countries. This allowed us to estimate the survey coverage for each country and better understand how representative the sample was.

The survey contained 34 questions with several of these including multiple answer options. To facilitate analysis, we adopted a stratified approach based on selected indicator questions. We acknowledge that this introduces an element of subjective judgement in determining estimation how best to present and summarize the results. However, we are confident that selection of other indicator questions would not have yielded qualitatively different results. We believe that this method provides a reliable descriptive overview of the current status of mspTBI care in European academic hospitals, further supported by the availability of complete response statistics for all questions in the supplementary material.

There are several different models of practice in the management of paediatric TBI, with significant variations, both between countries and, in some cases, within the same country. It is not clear how these variations affect the quality of care of this patient group. This survey was primarily conducted to chart the availability of dedicated mspTBI expertise and paediatric rehabilitation service across Europe. Accordingly, the organizational data obtained in the survey do not permit conclusions regarding the effect of such expertise on clinical outcomes. However, the findings provide a valuable framework for optimizing confidence and for future studies examining the relationship between patient outcomes and the access to dedicated mspTBI expertise.

## 5 Conclusion

This first European survey exploring the organizational management of mspTBI reveals low overall caseloads and uneven access to paediatric expertise even across academic centres. Confidence in managing mspTBI appears to correlate with the availability of paediatric subspecialists, suggesting that such expertise may serve as a surrogate indicator of quality of care. While guidelines are widely applied, regardless of expert availability, they alone appear insufficient to ensure clinician confidence in management. These findings underscore the need to strengthen both the specificity and practical applicability of guidelines, as well as to improve access to dedicated paediatric expertise in the management of mspTBI.

## Conflict of interest

The authors declare that they have no known competing financial interests or personal relationships that could have appeared to influence the work reported in this paper.
