# Supplementary material for: Organizational management of moderate and severe paediatric traumatic brain injury: results from a European survey
Source: Brain Spine. 2026 Jan 19;6:105921. doi: 10.1016/j.bas.2025.105921 (PMC12857177; doi:10.1016/j.bas.2025.105921)
Supplement: Multimedia component 1 [file mmc1.pdf]

Organization of Care for Pediatric Traumatic Brain Injury throughout Europe: a Multidisciplinary Survey

Introduction

Dear colleague

Pediatric traumatic brain injury (TBI) is a complex disease which requires specific expertise for management. As there is significant variation in the case-load of pediatric TBI across different European centers, there are several different models of practice in management of this disease. In order to identify best management pathways, we would like to map the needs and practice variations with this survey which is a joint initiative by the Trauma and Pediatric sections of the European Association of Neurosurgical Societies (EANS), the European Society of Pediatric and Neonatal Intensive Care (ESPNIC) and the European Society of Intensive Medicine (ESICM).
&
To maximally cover the field of involved specialists, the survey addresses:
- the general neurosurgery community in EANS member countries
- the pediatric intensive care community in ESPNIC member countries
- the general intensive care community in ESCIM member countries
&
The survey is divided in 7 modules: A. Hospital setting and resources; B. Management of pediatric TBI in your hospital; C. Who is in charge; D. Availibility of pediatric expertise; E. Rehabilitation and follow up; F. Registry; G. Your opinion.
&
Please know that information provided will be treated confidentially, without identification of particular institutes or physicians. Names of institutions are only asked to enable the grouping of information by different responders from within the same institution, after which they will be anonymized (It would therefore help if you go over the survey with your colleagues before submitting and submitting only one). It is crucial that data from this survey are based on reality; we will therefore ask you for honest and accurate replies. We wish to thank you in advance for your valuable input.
&
Yours sincerely
Shruti Agrawal (UK), Radek Fric (Norway), Chiara Robba (Italy), Aurelia Peraud (Germany), Marianne Juhler (Denmark), Miro Gjurasin (Croatia), Ondra Petr (Austria), Sarah Pedersen (Denmark), Bart Depreitere (Belgium)

Organization of Care for Pediatric Traumatic Brain Injury throughout Europe: a Multidisciplinary Survey

A. Questions regarding your hospital setting and resources

* 1. Please list the name of your hospital. This identifier is only intended to group responses of different respondents from the same hospital. After this processing, the hospital identifier will be removed and the rest of the analysis is anonymized.

[ ]

* 2. What country is your hospital located in?

[ ]

* 3. Is your hospital an adult hospital, pediatric hospital are a mixed hospital?

- ○ adult only
- ○ children only
- ○ mixed adults and children

* 4. How many neurosurgeons work as a staff in your hospital? Please express in full time equivalent.

[ ]

* 5. How many intensivists work as a staff in your hospital? Please express in full time equivalent.

[ ]

* 6. How many neurosurgeons are involved in pediatric neurosurgery as a major (more than half) part of their practice in your hospital?

[ ]

* 7. How many intensivists are involved in pediatric intensive care as a major (more than half) part of their practice in your hospital?

[ ]

* 8. To what intensive care facility are children with TBI admitted in your hospital?

- ○ dedicated pediatric intensive care unit with dedicated pediatric intensivist staff
- ○ dedicated pediatric intensive care unit with mixed pediatric and adult intensivist staff
- ○ mixed intensive care unit with mixed pediatric and adult intensivist staff
- ○ mixed intensive care unit with adult intensivist staff
- ○ adult intensive care unit with adult intensivist staff (only exceptionally admitting children)

9. If your hospital has a dedicated pediatric intensive care unit, what is the age range of children admitted to it?

* 10. Do **infants** with TBI in your hospital go to a (dedicated) neonatal ICU?

- ◯ yes
- ◯ no

11. If you answered 'yes' to question 10, please specify the age range that goes to neonatal ICU.

* 12. What size of population does your hospital approximately cover?

- ◯ < 250,000
- ◯ 250,000 - 500,000
- ◯ 500,000 - 1,000,000
- ◯ > 1,000,000

* 13. This hospital is

- ◯ academic
- ◯ non-academic
- ◯ mixed
- ◯ Other (please specify)

* 14. This hospital is

- ◯ public
- ◯ private
- ◯ mixed
- ◯ Other (please specify)

* 15. This hospital is a trauma hospital (i.e. includes a trauma service)

- ◯ level 1 (most comprehensive trauma care)
- ◯ level 2 (almost full coverage 24 hours)
- ◯ level 3 (no 24 hour and no full coverage)
- ◯ not applicable (levels do not apply in my country)
- ◯ Other (please specify)

B. Questions regarding the management of pediatric TBI in your hospital.

* 16. Does your hospital treat children with TBI? (more than 1 option can be ticked)

- [ ] yes, children with TBI are seen at the emergency department
- [ ] yes, children with TBI are admitted to regular wards
- [ ] yes, children with TBI are admitted to the ICU
- [ ] no

* 17. Are children with TBI brought to your hospital directly by ambulance services?

- ( ) yes
- ( ) no

* 18. Are children with TBI transferred to your hospital from other hospitals?

- ( ) yes
- ( ) no

19. If you answered 'yes' to question 18, please explain in what situations / for what reasons children with TBI are transferred from the other hospital(s).

* 20. Does your hospital transfer children with TBI to other hospitals?

- ( ) yes
- ( ) no

21. If you answered 'yes' to question 20, please explain in what situations / for what reasons children with TBI are transferred from your hospital to other hospitals.

* 22. How many children with all severities of TBI do you see per year approximately?

* 23. How many children with moderate/severe TBI do you see per year approximately?

* 24. How many **infants** (<1 year of age) with all severities of TBI do you see per year approximately?

* 25. How many **infants** with moderate/severe TBI do you see per year approximately?

* 26. How many children with non-accidental (abusive) head injuries do you see per year approximately?

* 27. What guidelines do you apply for TBI in children? (more than 1 option can be ticked)

- [ ] Scandinavian guidelines for initial management of minor and moderate head trauma in children, 2016
- [ ] Italian guidelines on the assessment and management of pediatric head injury in the emergency department, 2018
- [ ] Guidelines for the management of pediatric severe traumatic brain injury, 3rd edition, 2019, Brain Trauma Foundation
- [ ] The management of pediatric severe TBI: Italian guidelines, 2021
- [ ] national child-specific guideline
- [ ] Other (please specify)
- [ ] None

* 28. Do you use ICP monitoring in children?

- ( ) yes
- ( ) no

29. If you answered 'yes' to question 28: what type of ICP monitor device do you use? (more than 1 option can be ticked)

- [ ] EVD and pressure line
- [ ] parenchymal (microsensor, neurovent, camino etc)
- [ ] Other (please specify)

30. If you answered 'yes' to question 28: in what indications do you monitor ICP? Please include consciousness and CT scan criteria in your answer.

* 31. Do you use ICP monitoring in **infants**?

- ( ) yes
- ( ) no

32. If you answered 'yes' to question 31: what type of ICP monitor device do you use? (more than 1 option can be ticked)

- [ ] EVD and pressure line
- [ ] parenchymal (microsensor, neurovent, camino etc)
- [ ] Other (please specify)

33. If you answered 'yes' to question 31: in what indications do you monitor ICP? Please include consciousness and CT scan criteria in your answer.

* 34. What scoring method do you use in children for the assessment of consciousness? (more than 1 option can be ticked)

- [ ] Pediatric Glasgow Coma Scale
- [ ] Modified Glasgow Coma Scale for infants and children
- [ ] Child's Glasgow Coma Cale
- [ ] Regular Glasgow Coma Scale
- [ ] Other (please specify)
- [ ] None of the above

* 35. Are any advanced neuro-monitoring modalities available/used for children in your department? (more than 1 option can be ticked)

- [ ] continuous EEG
- [ ] non-continuous EEG (24/7 available)
- [ ] non-continuous EEG (in office hours)
- [ ] amplitude integrated EEG
- [ ] Near infrared spectroscopy
- [ ] Brain tissue oxygenation
- [ ] Microdialysis
- [ ] transcranial Doppler
- [ ] PRx
- [ ] other model based indices
- [ ] Other (please specify)

C. Who is in charge?

* 36. In your hospital, who is in-charge of management of pediatric TBI:

- general (adult) neurosurgeon
- pediatric neurosurgeon
- trauma surgeon
- general surgeon
- general (adult) intensivist
- neuro-intensivist (adult)
- pediatric intensivist
- pediatric neuro-intensivist
- team (different specialties manage together)
- different specialties are in charge in turn
- Other (please specify)

37. If you answered 'team' or 'different specialties in turn' to question 36, please explain who is part of this team or who takes charge in turn.

D. Availability of pediatric expertise in your hospital

* 38. Is there a 24/7 availability of pediatric neurosurgery expertise?

- no
- yes, effectively on call
- yes, can always be reached but not officially on call
- Other (please specify)

* 39. Is there a 24/7 availability of pediatric intensivist expertise?

- no
- yes, effectively on call
- yes, can always be reached but not officially on call
- Other (please specify)

* 40. Does your hospital have dedicated pediatric neuro-intensivists?

- yes
- no

* 41. Is there a 24/7 availability of pediatric neuro-intensivist expertise?

- no
- yes, effectively on call
- yes, can always be reached but not officially on call
- Other (please specify)

* 42. Does your hospital have pediatric anesthetists?

- yes
- no

* 43. Is there a 24/7 availability of pediatric anesthetist expertise?

- no
- yes, effectively on call
- yes, can always be reached but not officially on call
- Other (please specify)

E. Rehabilitation

* 44. What rehabilitation facilities are available in your set-up post discharge? (more than 1 option can be ticked)

- [ ] community based / primary care pediatric rehabilitation facility
- [ ] community based / primary care mixed adult/pediatric rehabilitation facility
- [ ] specialist pediatric rehabilitation facilities
- [ ] specialist mixed adult/pediatric rehabilitation facilities
- [ ] Other (please specify)

* 45. What follow up clinics do you use for children with TBI? (more than 1 option can be ticked)

- [ ] pediatric acquired brain injury clinic
- [ ] pediatric neurology clinic
- [ ] pediatric neurosurgery clinic
- [ ] mixed adult/pediatric acquired brain injury clinic
- [ ] mixed adult/pediatric neurology clinic
- [ ] mixed adult/pediatric neurosurgery clinic
- [ ] Other (please specify)

* 46. Do you use a specific time frame and frequency for follow-ups after TBI in children?

- ( ) yes
- ( ) no

47. If you answered 'yes' to question 46, please mention the time frame and frequency of folluw ups

F. Registry

* 48. Do you maintain a TBI registry in your set-up?

- [ ] no
- [ ] local
- [ ] regional
- [ ] national
- [ ] international
- [ ] Other (please specify)

* 49. Is there a national trauma registry in your country from which information on TBI can be retrieved?

- ( ) yes, pediatric only
- ( ) yes, adult only
- ( ) yes, general (all ages)
- ( ) no

G. Your opinion

* 50. In case you treat children with severe TBI in your hospital, do you feel comfortable managing these patients?

for all cases	for most cases	for about half of cases	for a minority of cases	for no cases	N/A
◯	◯	◯	◯	◯	◯

51. What help could you use to make you feel comfortable in all cases of severe TBI? (more than 1 option can be ticked)

- ☐ specific guidelines for management
- ☐ regionally organized stepped referral system
- ☐ tele-consulting experienced pediatric neurosurgeons/intensivists
- ☐ not applicable (our hospital is fully equipped and has a high expertise)
- ☐ Other (please specify)

* 52. In case you treat **infants** (less than 1 year of age) with moderate/severe TBI in your hospital, do you feel comfortable managing these patients?

for all cases	for most cases	for about half of cases	for a minority of cases	for no cases	N/A
◯	◯	◯	◯	◯	◯

53. What help could you use to make you feel comfortable in all **infants** with moderate/severe TBI? (more than 1 option can be ticked)

- ☐ specific guidelines for management
- ☐ regionally organized stepped referral system
- ☐ tele-consulting experienced pediatric neurosurgeons/intensivists
- ☐ not applicable (our hospital is fully equipped and has a high expertise)
- ☐ Other (please specify)

* 54. Children with severe TBI

- ◯ Should always be transferred to a center with higher volume and more experience
- ◯ Should receive life-saving surgery and then be transferred to a center with higher volume and more experience
- ◯ All neurosurgery centers should be able to manage children with TBI
- ◯ Other (please specify)

55. Please enter free comments below, in case you still want to add personal views.

Organization of Care for Pediatric Traumatic Brain Injury throughout Europe: a Multidisciplinary Survey

We sincerely thank you for your input!

56. Please leave your name/email address if you are happy for us to contact you directly for further information.
